# Protoplast Technology and Somatic Hybridisation in the Family Apiaceae

**DOI:** 10.3390/plants12051060

**Published:** 2023-02-27

**Authors:** Ankush S. Ranaware, Nandkumar S. Kunchge, Smita S. Lele, Sergio J. Ochatt

**Affiliations:** 1 Institute of Chemical Technology, Marathwada Campus, Jalna 431203, Maharashtra, India; 2 Research and Development Division, Kalash Seeds Pvt. Ltd., Jalna 431203, Maharashtra, India; 3 Agroécologie, InstitutAgro Dijon, INRAE, Université Bourgogne Franche-Comté, 21000 Dijon, France

**Keywords:** biotechnological breeding, carrot, celery, cybrids, male sterility, protoplast isolation and fusion

## Abstract

Species of the family Apiaceae occupy a major market share but are hitherto dependent on open pollinated cultivars. This results in a lack of production uniformity and reduced quality that has fostered hybrid seed production. The difficulty in flower emasculation led breeders to use biotechnology approaches including somatic hybridization. We discuss the use of protoplast technology for the development of somatic hybrids, cybrids and in-vitro breeding of commercial traits such as CMS (cytoplasmic male sterility), GMS (genetic male sterility) and EGMS (environment-sensitive genic male sterility). The molecular mechanism(s) underlying CMS and its candidate genes are also discussed. Cybridization strategies based on enucleation (Gamma rays, X-rays and UV rays) and metabolically arresting protoplasts with chemicals such as iodoacetamide or iodoacetate are reviewed. Differential fluorescence staining of fused protoplast as routinely used can be replaced by new tagging approaches using non-toxic proteins. Here, we focused on the initial plant materials and tissue sources for protoplast isolation, the various digestion enzyme mixtures tested, and on the understanding of cell wall re-generation, all of which intervene in somatic hybrids regeneration. Although there are no alternatives to somatic hybridization, various approaches also discussed are emerging, viz., robotic platforms, artificial intelligence, in recent breeding programs for trait identification and selection.

## 1. Introduction

The rapidly growing population has made crop improvement for enhancing yield and quality to center stage, in order to serve the ever-increasing food needs. Since the onset of crop improvement, almost ten thousand years ago, selection and breeding have been used routinely and continuously for crop improvement, as stressed by the late eminent plant molecular biologist, Lawrence Bogorad from Harvard University [1]. The limited range of genes accessible through conventional sexual breeding within crossable plants species has hindered the introduction of genes or traits of interest. Thus, for many crops a long period is required for the purification and selection process for a desired trait or gene in a new genotype. Technology advancement in the manipulation of cells in vitro and genetic engineering offer an alternative to conventional plant breeding and provide novel approaches for gene pooling that hitherto were not available naturally in the environment [2].

At present, the role of agriculture in ensuring food security is strategic to meet the increasingly growing population demand and simultaneously address the very many up-surging environmental challenges resulting from global warming [3]. According to the most recent United Nations estimates (https://www.worldometers.info/; accessed on 3 February 2023), the world population reached 7.93 billion in March 2022. However, it would more likely exceed 9 billion according to an AsiaNews (PIME) report that already in 2017 about a billion people around the world had no identities and were hence invisible. Still, their need for food adds to the huge demand that already exceeds the current production globally (https://www.asianews.it/news-en/About-a-billion-people-are-invisible,-one-third-of-them-children-42131.html) (accessed on 9 December 2022). It is therefore necessary to rapidly boost food output without expanding agricultural lands but also using less water, fertilizers, pesticides and herbicides per cultivated hectare in order to reduce emissions from production processes and environmental pollution.

**Figure 1:**
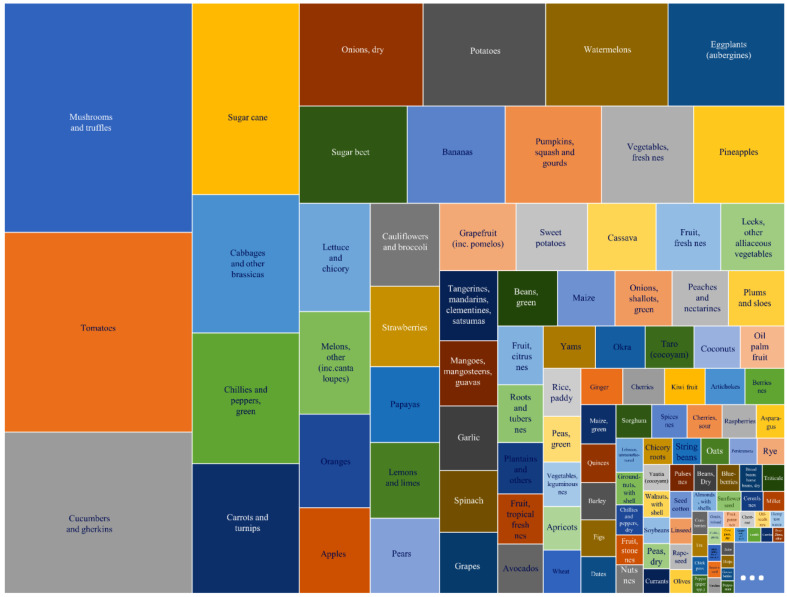
Yield data of primary crops of the year 2020 from Food and Agriculture Organization of the United Nations (https://www.fao.org/faostat/en/#data/QCL) (accessed on 10 February 2022).

Crops of importance for our diet under cultivation on the planet cover different surfaces and produce variable yields (Figure 1). Amongst them, open-pollinated cultivars are majoritarian in the market for members of the family Apiaceae, such as carrot (*Daucus carota*), celery and celeriac (*Apium graveolens* var. *dulce* and var. *rapaceum*, respectively). Such cultivars, although productive, often lack uniformity and yield regularity and these problems may be resolved by developing and using hybrid seeds. The availability of high-quality male sterile lines is of paramount importance in this respect and remains currently a bottleneck for hybrid breeding of crops in the family Apiaceae. 

In this respect, male sterility results from the failure to produce either dehiscent anthers, functional pollen, and/or viable gametes. Its evolutionary importance has long been recognized but its breeding utility was initially ignored, until the breeding potential of hybrid vigor as a means of studying the influence of cytoplasm on plant development was acknowledged. However, if the offspring of any cross between two genetically different plants can be considered to be a hybrid, the process is not that simple. Indeed, in self-fertile crops it requires emasculation of flowers, which is laborious and costly or, when performed chemically, is environmentally unfriendly. Hence the great interest of male-sterile genotypes for such crops [4].

In this review, we focus particularly on both genetic (GMS) and cytoplasmic (CMS) male sterility [4,5], on the candidate genes involved in the process, and on the production of such male sterile genotypes through somatic hybridization. Thus, we review the pre-treatments of donor plants, the different tissues used as sources of the parental protoplasts, the methods for obtaining somatic hybrids and cybrids as well as their subsequent culture. Crucial steps for understanding cell wall regeneration in species of the family Apiaceae are also discussed.

## 2. The Role of Male Sterility in Plant Breeding

There are two sources of male sterility, genetically encoded (GMS) and cytoplasmic (CMS), which bypass the time-consuming work of flower emasculation in umbels of crops in the family Apiaceae. GMS is determined only by genes encoded in the nuclear genome [4], while CMS is due to mitochondrial genes that affect nuclear gene functions directly or indirectly.

## 3. Genetically Encoded Male Sterility (GMS)

**Figure 2:**
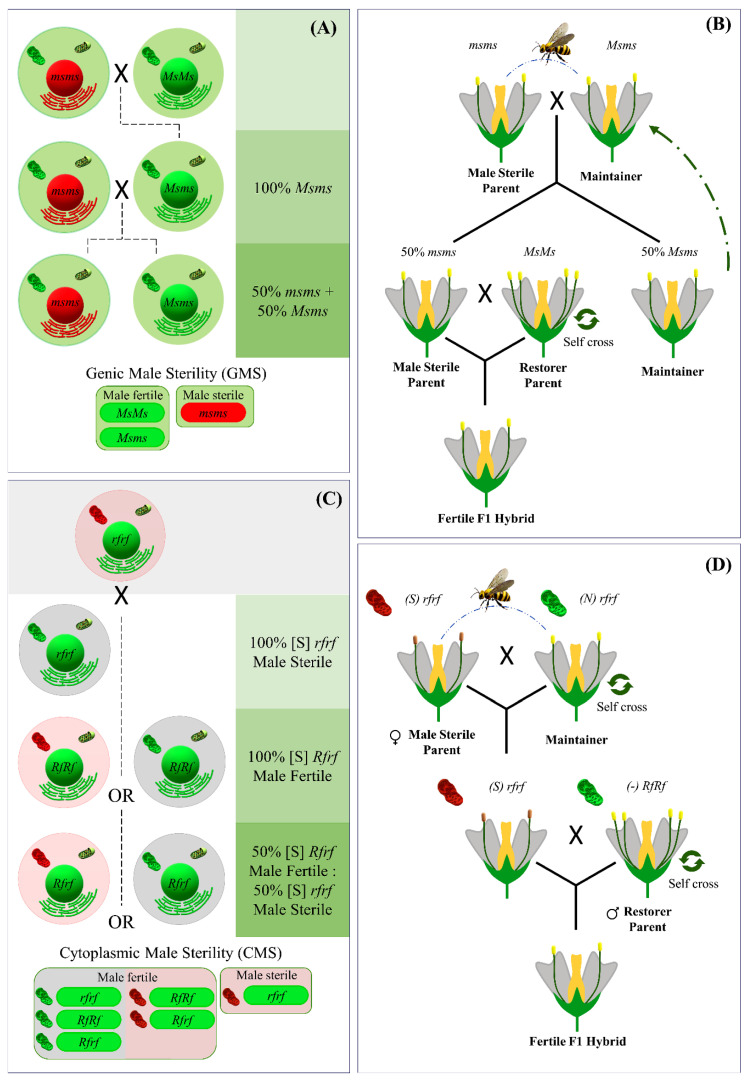
(**A**) Simple genetic model of Genic male sterility (GMS), (**B**) Maintenance of parent line and hybrid seed production for GMS, (**C**) Simple genetic model of Cytoplasmic Male Sterility (CMS), (**D**) Maintenance of parent line and hybrid seed production for CMS.

GMS is under the control of various nuclear male sterility genes that are not influenced by cytoplasmic sequences. As a result of this, in a simple genetic situation with a Mendelian inheritance pattern there will be three possible genotypes for the nuclear locus *MS* (Male Sterility), where the male sterile phenotype is determined by the allele at its recessive (*ms*) status (Figure 2). Therefore, for such a male sterile genotype (which in breeding would represent the female line), the offspring may be 100% male fertile (for a parental line homozygous for the nuclear restorer-of-fertility locus) or segregate 1:1 yielding 50% male sterile: 50% male fertile plants (for a heterozygous parent). 

Using GMS for breeding requires availability of a male sterile line (the female parent), a maintainer line (isogenic but for the presence of a dominant *MS* allele), and a restorer line (the male parent, which has dominant restorer-of-fertility alleles *Rf* and thus produces fertile F1 hybrids). In this context, the segregation in the cross with the maintainer line introduces an additional selection step among male sterile phenotypes to remove all heterozygotes for the production of hybrid seeds, which has limited the use of GMS in crops where CMS is not available. Cytoplasmic male sterility (CMS) has been observed in more than 300 species to date [5]. This laborious practice of handling a male sterile line, a maintainer line and a restorer line simultaneously in the GMS system can be overcome by environment-sensitive genic male sterility (EGMS). Thus, in rice 5 to 10% higher yield is obtained with respect to land area based on EGMS system. EGMS plays a crucial role in selection of male sterility dependent on sensitivity and adaption to the environmental factors such as high or low temperature, humidity and photoperiod [6]. In a rapeseed TGMS (thermosensitive genic male sterile) line, a temperature above 20 °C resulted in an alteration of gene expression due to a defect of homologous pairing in meiosis I [7]. 

In wheat, a TGMS line was fertile at 20 °C and sterile at 10 °C as a result of miRNA differential expression [8]. Recently, Zhu and colleagues hypothesized that in an Arabidopsis TGMS line low temperature slowed down pollen development which in turn helped in restoring fertility of the thermo-sensitive male sterile plant [9]. 

## 4. Cytoplasmic Male Sterility (CMS)

CMS, being maternally inherited, is the preferred material since it permits a better control of hybrid seed production [10]. It has been increasingly applied to a number of major cereal, vegetable, legume, oilseed, industrial and ornamental crops, including various members of the family Apiaceae on which this review is focused. However, it must be stated that in general the hybrids developed have a narrow genetic base due to the use of a very limited number of sources of CMS in plant breeding. Therefore, future research in this domain should concentrate on widening the range of cytoplasmic genes that produce male sterile phenotypes as also their respective nuclear-encoded restorer-of-fertility genes. The recent advent of new genetic tools to study the dynamics of the mitochondrial genome as well as its interaction with the nuclear genes offers novel experimental frameworks to efficiently address these challenges [11,12]. 

CMS phenotypes have often arisen in wild populations through spontaneous mutations involving rearrangements of the mitochondrial genome (mtDNA), which resulted from intragenomic homologous or non-homologous recombination events and created new open reading frames (ORFs) [13]. Disruption of the nuclear gene *Msh1* by a transgenic RNAi approach in both tomato and tobacco led to stoichiometry in plant mitochondria which allowed expression of the CMS phenotype ORFs which were at low copy number [14]. Yet, in onion the existence of the CMS ORF in fertile lines at extremely low concentrations plays an important role for the expression of ORF [15]. A full understanding of the molecular mechanisms underlying the CMS condition remains difficult because of its uniqueness in terms of the mitochondrial genes associated with the male sterility condition [16]. Furthermore, there are, for instance, CMS lines frequently used in rice breeding where a specific restorer line was developed but the identity of the gene responsible for the male sterility condition is still not known [17,18]. 

Figure 2 also depicts a scheme of the genetic model of CMS. 

Mitochondrial dysfunction is caused by nuclear MADS-box genes that control whorls 2, 3 and even whorl 1. Out of 16 MADS-box genes, *DcPI* and *DcAG*-like genes showed a significantly lowered expression in petaloid CMS lines. Among 12 differentially expressed genes of oxidative phosphorylation, the two genes *DcNad2* and *DcNad5*, which encode NADH dehydrogenase had a significantly lower expression in a petaloid CMS line of carrot as compared to the maintainer line. This led to the formation of a petaloid or carpeloid CMS system, due to energy deficiency in mitochondria which constitute the cell power-house [19]. This shows a larger energy requirement of reproductive organs compared to other organs. Likewise, in species of other families e.g., wild beet, constitutive mRNA expression of *orf129* of CMS protein was observed in flowers, leaves and roots, but it only affected the anther development and led to male sterility. In maize, too, CMS-T protein also governs cytotoxic activity resulting in male sterility, while it is also toxic to *Escherichia coli* and eukaryotic cells. Thus, to date, CMS proteins were reported in sunflower, radish, rice, *Brassica* sp., among others [4]. 

Programmed cell death (PCD) is a necessary developmental event that regularly happens in animals and plants. For plants, it involves developmental processes such as seed germination, root tip elongation, xylem and aerenchyma formation, organ development, senescence and disease resistance, among others [20]. PCD also plays an important role in CMS development through a co-operative interaction between the anther wall and the microspores. Such interaction requires the cellular degradation of the innermost cell layer of the anther (tapetum) which surrounds the microspores [21]. In a carpeloid CMS line of carrot, the expression of two nuclear located MADS-box genes *DcMADS2* and *DcMADS3* was suppressed in developing flowers of whorls 2 and 3, which are homologues to *GLOBOSA* and *DEFICIENS* of the *Antirrhinum* genes [22]. In *Citrus*, expression analysis found an involvement of miRNAs targeting transcription factors involved in floral development of *Citrus grandis* (male sterile cybrid pummelo), such as auxin response factors (*ARFs*), *MYB*, *SQUAMOSA* promoter binding protein box (SBP-box), *APETALA2* (*AP2*), basic region-leucine zipper (*bZIP*) and transport inhibitor response 1 (*TIR1*). This indicated a strong interaction between mitochondrial CMS genes and nuclear MADS-box genes, while nuclear-cytoplasmic retrograde signaling must be playing an important role in CMS flower development through miRNA expression as well [23]. 

Alternatively, a fertile line and a CMS line can be combined through somatic hybridization to give a male-sterile plant with a chimeric mitochondrial genome, as the limited homologous recombination yields a mtDNA with few regions from the CMS parent, whereby candidate genes for CMS may be proposed [24]. Conversely, if mtDNA rearrangements generate numerous new ORFs, a differential expression assay and/or a segregation analysis will be required for the identification of the CMS candidate [25]. On the other hand, some CMS systems have recently been linked to intron splicing and RNA editing, where cytidines are changed to uridines in specific editing sites located in diverse positions of mitochondrial mRNAs [13]. This alters organellar protein products, and renders RNA editing essential as it allows the synthesis of functional organellar proteins crucial for plant and seed development [26]. Thus, impairing mitochondrial function through a deficient RNA editing induces the synthesis of abnormal proteins that, in turn, may induce male-sterility. Interestingly, the exact relationship between the candidate CMS gene and the observed phenotype has been assessed rarely, while the mechanisms of action of the restorer-of-fertility loci remain poorly understood. Comparing directly the mtDNAs, although difficult in such highly rearranged genomes, could help in identifying the gene(s) responsible for the CMS condition [15].

## 5. Cytoplasmic Male Sterility in the Family Apiaceae 

In the family Apiaceae, the Iranian accession ‘P1229526′ is the first male sterile line in celery and was reported by Quiros et al. in 1986 [27]. In two successive publications, Gao et al. first attributed CMS to a *ms-1* single recessive gene in 2006 [28]. However, in 2009 they concluded that in fact two recessive genes controlled nuclear male sterility [29]. Recently in the Chinese celery “tanzhixiangqin”, comparative analysis of the mitochondrial genome between the CMS line and its maintainer line opened the door to identifying 21 unique regions with 15 ORFs in the CMS line. Only one chimeric gene was found in ORF768a that had a 1497 bp sequence of the *cox1* gene and an unknown sequence of 810 bp. Probably, ORF768a coded for the 11 transmembrane domain of protein, which led Cheng et al. [30] to indicate in 2021 that *ORF768a* might be a good candidate gene for cytoplasmic male sterility in celery. In carrots, a petaloid CMS line was associated with a novel ORF of 651 bp (*orfB-CMS*) and an additional 170 bp unknown sequence. This *orfB-CMS* chimeric gene has a functional role in *atp8*-like membrane protein [4,31]. Unfortunately, the number of stable male sterile lines available in species of the family Apiaceae remains limited [27,28], and none is presently used for commercial seed mass production. However, the need for growth uniformity is testified by the increasing number of hybrid cultivars available in the market despite the lack of an efficient CMS system. 

## 6. Protoplast Technology in the Family Apiaceae

Compared to sexual reproduction, somatic hybridization has many advantages to transfer or generate the CMS condition de novo, in particular because it avoids unwanted/uncontrolled traits coming from the simultaneous transmission of genes other than those responsible for CMS. However, a number of prerequisites exist for the successful exploitation of somatic hybridization in this context. They include an efficient and reliable isolation of large yields of highly viable protoplasts of both partners, and the establishment of reproducible strategies for the high frequency regeneration of plants from the cultured protoplasts of at least one of the prospective fusion partners. This must also be coupled with efficient procedures for obtaining viable heterokaryons after protoplast fusion that, in turn, will be competent for division and ultimate plant regeneration. The information available on these various steps for members of the family Apiaceae will be discussed in the sections below.

## 7. Protoplast Isolation from Different Tissue Sources in Species of the Family Apiaceae

In 1880, Hanstein first coined the term protoplast representing the entire cell without its wall. Thereafter, Klercker (1892) for the first time used mechanical means to obtain protoplasts from water warrior (*Stratiotes aloides*) [32]. The pioneering discovery by Prof. Edward Cocking in 1960 [33] that protoplast isolation could be achieved by enzymatic methods led to gigantic progress in the engineering of somatic cell genetics by making easier the entry of foreign genes into cells lacking the cell wall. 

To date, isolation of protoplasts was reported in the family Apiaceae in *Daucus carota* (Carrot), *Apium graveolens* (Celery), *Coriandrum sativum* (Coriander), *Foeniculum vulgare* (Fennel), and *Petroselinum Hortense* (Parsley) (see Reference Table 1) [34,35,36,37,38]. A wide variety of tissues and organs have been used for isolation of protoplasts, viz., leaves, petals, petioles, cotyledons, shoot apices, epicotyl, hypocotyls, coleoptiles, stems, embryos, microspore mother cells, microspore tetrads, pollen grains, tubers, roots, root nodules, fruits, endosperm, aleurone layer, crown-gall tissue, callus and cell suspension cultures [32,35,39,40]. Plants grown in the field, in a greenhouse and aseptically in vitro are the materials routinely used as source tissue for the isolation of protoplasts. They have their own limitations and benefits, as the use of source tissues grown in a controlled environment and physiological state influences both the yield and the viability of isolated protoplasts. Greenhouse-grown crops are often available but they are dependent on temperature, light and supply of nutrients which are not always controlled enough for optimum growth. Environmentally controlled chambers help in reducing the associated variation of greenhouse and field-grown plants [39]. Different enzyme mixtures have been used to date along with combinations of various concentrations for obtaining better yield (see References Table 1 and Table 2). In the case of both carrot [41] and cabbage [42], leaf-derived protoplasts gave yields three times those of hypocotyl protoplasts. In this respect, most authors preferred to use cell suspensions as the protoplast source rather than differentiated tissues such as with leaves or hypocotyls of celery [43]. In non-Apiaceae species like Sowbread cyclamen, embryogenic cell suspensions gave higher yields compared to somatic embryos [44]. *Alstroemeria* friable embryogenic callus was found to be a better source of protoplasts than leaves or compact embryogenic callus, maybe due to the exposure of a larger surface area to the enzyme solution, resulting in better digestion [45]. Similarly, for the silk tree, leaves digested with 1.5% cellulase gave higher protoplast yields compared to hypocotyl-derived callus although these were digested with a higher (2%) cellulase concentration, and this was coupled with higher viability (of 87% and 85%, respectively) even if enzyme digestion time was 6 h and 16 h, respectively [46]. In *Brassica oleracea*, selection of the tissue source and age of explant was one of the important steps in protoplast isolation, and 4 to 6 week old leaf explants and 1 week old hypocotyls gave the highest protoplast viability compared to 2 week old tissues of either source [42]. 

For a successful isolation of viable protoplasts, pre-treatments applied to the source tissues before enzyme digestion play a crucial role. These may include both a physical disruption (i.e., chopping of tissues) and/or coupling with a pre-plasmolysis in a solution consisting of either sucrose or metabolically inert sugars, among which mannitol and sorbitol are often preferred [44,47,48]. Interestingly, in carrot, plating efficiency of hypocotyl protoplasts was twice that of leaf protoplasts, while the routinely used suspension culture-derived protoplasts exhibited a plating efficiency even lower than that of those two differentiated tissue sources [41].

**Table 1:** Protoplast isolation in the family Apiaceae [36,41,49,50,51].

Plant Genotypes (*Daucus carota* = *Dc*)	Tissue Source	Enzyme Mixture and Condition	Yield and Viability	References
Carrot	Leaves and Hypocotyls	1% Cellulase Onozuka R10, 0.1% Pectolyase Y-23, 0.6 M Mannitol, 5 mM CaCl_2_, 20 mM MES, 14–18 h, 30 rpm, 26 °C	Leaves: 3.21 × 10^6^ gfw^−1^, 74% Viability; Hypocotyls: 0.96 × 10^6^ gfw^−1^	[41]
	Leaves	1% Cellulase Onozuka R10, 0.1% Pectolyase Y-23, 0.6 M Mannitol, 5 mM CaCl_2_, 10 mM MES, 14–16 h, Dark, 30 rpm, 26 °C	Not reported	[49]
	Leaves	1% Cellulase Onozuka R10, 0.1% Pectolyase Y-23, 0.6 M Mannitol, 5 mM CaCl_2_, 20 mM MES, 12–16 h, Dark, 30 rpm, 26 °C	2.8 × 10^6^ gfw^−1^, 72–93% Viability	[50]
	Leaves	2% Cellulase Onozuka R10, 0.1% Pectolyase Y-23 and 1% Macerozyme R-10, 0.6 M Mannitol, 10 mM CaCl_2_, 10 mM MES, 0.8% Bovine serum albumin, 15 h, Dark, 30 rpm, 26 °C	Not reported	[51]
Coriander (*Coriandrum sativum* vars.)	Embryogenic cell suspension	2% Cellulase Onozuka R10, 1% Pectinase and 0.2% Macerozyme R-10, 0.6 M Mannitol, 5 mM CaCl_2_, 14–18 h, Dark, 50 rpm	4.81 × 10^6^ gfw^−1^, 90–93.8% Viability	[36]

**Table 2:** Examples of protoplast fusion and somatic hybridization with the family Apiaceae [2,34,38,52,53,54,55,56,57,58,59,60,61,62,63,64,65,66,67,68,69,70,71,72,73,74,75,76,77,78,79].

Plant Genotypes (*Daucus carota* = *Dc*)	Tissue Source ^a^	Enzyme Mixture and Condition	Pre-Treatment ^b^	Chemical/Electrofusion	Fusion Type	References
*Dc*	CS	2% Driselase, 0.4 M Sorbitol (2–3 h)	-		S	[52]
*Dc* × *Hordeum vulgare L*	CS × L	4% Onozuka P 1500, 1% Driselase, 1% Pectinase, 1% Rhozyme, 0.35 M Sorbitol and 0.35 Mannitol, 6 mM CaCl_2_.2H_2_O, NaH_2_PO_4_.H_2_O, 3 mM MES (Mixed with Equal Volume of CS) (4 h, 50 rpm, 26 °C)	-	Chemical	HK/S	[53]
*Hordeum vulgare* L., cv. Taplavni tavaszi × (*Dc* L., cv. Nantaise Slender)	L × CS	4% Onozuka P 1500, 1% Driselase, 1% Pectinase, 1% Rhozyme, 0.35 M Sorbitol and 0.35 Mannitol, 6 mM CaCl_2_.2H_2_O, NaH_2_PO_4_.H_2_O, 3 mM MES (Mixed with Equal Volume of CS) (5 h, 50 rpm, 25 °C)	-	Chemical	HK/S	[54]
(*Dc*) × *D. capillifolus*	CS × CS	4% Onozuka P 1500, 1% Driselase, 1% Pectinase, 1% Rhozyme, 0.35 M Sorbitol and 0.35 Mannitol, 6 mM CaCl_2_.2H_2_O, NaH_2_PO_4_.H_2_O, 3 mM MES (Mixed with Equal Volume of CS) (4 h, 50 rpm, 26 °C)	-	Chemical	HK/S	[55]
*Dc* L. (albino) × *Aegopodium podagraria*	CS × L	4% Onozuka P 1500, 1% Driselase, 1% Pectinase, 1% Rhozyme, 0.35 M Sorbitol and 0.35 Mannitol, 6 mM CaCl_2_.2H_2_O, NaH_2_PO_4_.H_2_O, 3 mM MES (Mixed with Equal Volume of CS) (4 h, 50 rpm, 26 °C)	-	Chemical	HK/S	[56]
*Dc* × *Petroselinum Hortense*	CS × L	4% Onozuka P 1500, 1% Driselase, 1% Pectinase, 1% Rhozyme, 0.35 M Sorbitol and 0.35 Mannitol, 6 mM CaCl_2_.2H_2_O, NaH_2_PO_4_.H_2_O, 3 mM MES (Mixed with Equal Volume of CS) (16 h, 4 h for Parsley, 50 rpm, 26 °C)	X-irradiation of 9kR (Parsley)	Chemical	HK/A	[38]
*Dc* (C123) × *D. capillifolius*	CS R line × CS Seedling	0.1% Macerozyme R-10, 0.1% Cellulase R-10, 10% Mannitol, 0.1% CaCl_2_.2H_2_O (12 to 24 h, 25 °C)	-	Chemical	HK/S	[57]
*Dc* L. var. Danvers (Line C81) × (line C123)	CS both	2% Cellulase Onozuka-R10, 1% Macerozyme R10, 1% Driselase purum, 0.4 M Mannitol, 10 mM CaCl_2_, 0.5% MES (4.5 h, 25 °C)	-	Chemical	HK/S	[58]
(*Dc* L. cv. Lunga di Amsterdam) (C1) × (*Dc* A2CA-N)	CS both	2% Cellulase Onozuka R-10, 1% Macerozyme, 0.4 M Mannitol (4 h, 25 °C, gentle shaking)	IOA 0.2 mM and incubated at 25 °C for 20 min	Chemical	HK/S	[59]
*Spinacia oleracea* L. (cv. Hybrid 102) × *Dc* L. (cv. Western Red)	L × R	4% Onozuka Cellulase R-10, 0.1% Pectinase, 0.8 M Mannitol, 7 mM CaCl_2_ (16–20 h, Dark, 25 °C, 25 rpm)	-	Chemical	HK/S	[60]
(*Dc* L. cv. Lunga di Amsterdam) × rice (*Oryza sativa* L. cv. Roncarolo)	CS (Carrot R) × Rice (seedling callus)	1% Cellulase Onozuka RS, 2% Pectinase, 0.4 M Mannitol (5 h, 26 °C, gentle shaking)	-	Chemical	HK/S	[61]
*Dc*	ECS	2% Driselase, 0.4 M Sorbitol, 2.5 mM EGTA, 1 mM MES (2 h)	Carboxyfluorescein, Scopoletin, FITC, RITC, RHO 123, RHO B ethyl ester	Chemical	HK/S	[62]
*Dc*	ECS	2% Driselase, 0.4 M Sorbitol, 1 mM MES (2 h, 125 rpm, 25 °C)	-	Chemical	HK/S	[63]
*Dc*	ECS	2% Driselase, 0.4 M Sorbitol, 2.5 mM EGTA, 1 mM MES (2 h, 125 rpm, 25 °C)	fluorescent dyes (carboxyfluorescein, RHO 123, and RHO B ethyl ester)	Chemical	HK/S	[64]
*Dc*	CS	2% Driselase, 0.4 M Sorbitol, 1 mM MES	1 mM DFMA final concentration (Inhibitor)	Chemical	HK/S	[65]
*Dc* L. cv. Lunga di Amsterdam (A2CA^r^-N × E2A1)	CS	2% Cellulase Onozuka R-10, 1% Macerozyme, 0.4 M Mannitol (4 h, 25 °C, gentle shaking)	-	Chemical	HK/S	[66]
*Dc* L. X *D. capillifolius* Gilli	CS (H derived calli) × CS	1% Driselase, 0.5% Cellulase Onozuka RS, 0.01% PectolyaseY-23, 0.5 M Mannitol, 0.1% MES (4 h, 25 °C, Occasional Shaking)	(15 mM IOA, 10 min at RT) × (X-irradiated of 60 Krad)	Chemical	HK/Cybrids	[67]
*N. plumbaginifolia* Viviani (NX1) × *Dc* cv. Danvers (strain, PR)	L × R	2% Cellulase Onozuka R10, 0.1% Macerozyme, 10% Mannitol, 0.1% CaCl_2_.2H_2_O (14–16 h, 30 rpm)	-	Chemical	HK/S	[68]
*Dc*	CS	2% Driselase, 0.4 M Mannitol (5 h)	-	Electrofusion	HK/S	[69]
*Dc* × (*Nicotiana tabacum*)	CS × L	2% Cellulase R-10 or 2% Cellulysin, 1% Pectinase, 0.5% Pectinase, 0.5% Driselase, 0.5% Rhozyme, 0.35 M Mannitol, 0.35 M Sorbitol, 3 mM MES, 6 mM CaCl_2_.2H_2_O, 0.7 mM NaH_2_PO_4_ (Overnight, 25 °C, 50 rpm)	53 Gy, 18%; 107 Gy, 2%; 166 Gy, 0.5%. X Non-irradiated	Chemical	HK/A	[70]
*Dc* 28K CMS line × *Dc* cv. Kikuyo gosun (K5)		1% Driselase, 0.5% Cellulase Onozuka RS, 0.01% PectolyaseY-23, 0.5 M Mannitol, 0.1% MES (4 h, 25 °C, Occasional Shaking)	X-irradiated with a total dosage of 60 Krad (1 Krad/min) × 15 mM IOA for 10 min.	Chemical	Cybrids	[71]
*Dc* CMS line, 28A1 (brown anther type) × fertile *Dc* cultivar ‘K5’	CS × H	1% Driselase, 0.5% Cellulase Onozuka RS, 0.01% PectolyaseY-23, 0.5 M Mannitol, 0.1% MES (4 h, 25 °C, Occasional Shaking)	X-irradiated at 60 Krad (1 Krad/min) × 15 mM IOA	Chemical	HK	[72]
*D. capillijolius* and *Dc* ssp. Gummijer × *Dc* cv. NS and 35B	CS	1% Driselase, 0.5% Cellulase RS, 0.01% Pectolyase Y-23, 0.5 M Mannitol, 0.1% MES (1–6 h, 25 °C, Shaking)	X-irradiated with a total dosage of 60 Krad (1 Krad/min) × 15 mM lOA	Chemical	Cybrids	[2]
*Nicotiana tabacum (KR-SR)* × *Dc L. and Nicotiana tabacum (KR-SA)* × *Dc L.*	L × CS and CS × CS	1.6% Cellulase Onozuka R-10, 0.3% Macerozyme R10, 8% Mannitol, 0.1% CaCl_2_.2H_2_O (3 h, 25 °C)	Irradiated with 7 Krad or 10 Krad of X-rays	Electrofusion	Somatic hybrid	[73]
*Dc L.* × *Oryza sativa L.*	CS	1.6% Cellulase Onozuka R-10, 0.3% Macerozyme R10, 8% Mannitol, 0.1% CaCl_2_.2H_2_O (3 h, 25 °C)	50 Krad X-rays × 10 mM IOA for 20 min	Electrofusion	HK/A	[74]
*Barley (Hordeum vulgare L.)* × *Dc L.*	L × CS	1.6% Cellulase Onozuka R-10, 0.3% Macerozyme R10, 8% Mannitol, 0.1% CaCl_2_.2H_2_O (3 h, 25 °C)	_	Electrofusion	HK/A	[75]
*Daucus carota ssp. sativus (Hoffm.) Arcang.* (Eight fertile cultivars × MS-1 CMS line)	CS young L or H	1% Driselase, 0.5% Cellulase RS, 0.01% Pectolyase Y-23, 0.5 M Mannitol, 0.1% MES (3 h, 30 °C, 50 rpm)	Irradiated with 85 Krad X-ray × 15 mM IOA for 20 min at 4 °C	Electrofusion	Cybrids	[76]
Celery (*Apium graveolens* L.) × CMS *Dc*			6 mmol.L^−1^ IOA for 7 min × irradiated with UV rays (20 µmol.m^−2^.s^−1^) for 9 min	Chemical	HK/A	[77]
*Dc var. sativus Hoffm.* × *American ginseng (P. quinquefolius L.)*	CS × calli	1.5% Cellulase Onozyka RS, 0.3% Pectolyase Y-23, 0.6 M Mannitol, 5 mM CaCl_2_ (4 h, RT, 70 rpm)	-	Chemical	HK/A	[78]
*Dc* ssp. *sativus* Hoffm. × Amsterdamska (A) and Koral (K)	CS	1% Cellulase Onozuka R-10, 0.1% Pectolyase Y-23, 0.6 M Mannitol, 5 mM CaCl_2_, 20 mM MES (14–18 h, Dark, 30 rpm, 26 °C)		Electrofusion	Homo-fusant/ Homo-fusant	[79]
‘Diamant’ celeriac (acceptor) and ‘Parmex’ carrot, coriander or ‘WL253’ white celery (donors)	CS × L and P	1.5% Cellulase R10, 0.1% Macerozyme (Overnight, Dark, 30 rpm, 22 °C)	10 mM IOA for 20 min × UV (257 µWcm^−2^) for 6 min	Electrofusion	Hetero-fusant	[34]

^a^ Tissue sources, CS: Cell Suspension, ECS: Embryogenic Cell Suspension, H: hypocotyl, L: leaf, P: Petiole, R: root; ^b^ IOA: iodoacetamide, RHO: Rhodamine, S: Symmetric, A: Asymmetric, HK: Heterokaryon.

## 8. Understanding Cell Wall Regeneration for an Improved Efficiency of Protoplast Technology in Carrot

The key to an efficient exploitation of protoplast technology for breeding includes a necessary step of de novo regeneration of the cell wall after protoplast isolation. Knowledge on this fundamental stage has been the object of several studies for many years, including in carrot which has, in this respect, sometimes even been considered as a model system.

Thus, in an early work, Shea et al. [80] examined the structure of the cell wall regenerated by carrot protoplasts for a better understanding of the mechanisms contributing to their subsequent viability and division competence in culture. They found that callose, one of the (1 → 3)β-d-glucan fractions that constitute hemicelluloses whose synthesis is generally associated with tissue and cell wounding, was not a component of the incipient wall of carrot protoplasts. Conversely, callose synthesis could be induced by intentional wounding, while the acid-resistant cellulose was formed more slowly. As a result, the complete regeneration of the wall required 3 days and the resulting protoplast-derived cell was unable to cope with turgor before at least 5 days. On the other hand, the pectic substances synthesized by protoplasts were less anionic than those of parent cells, and they became more highly charged during wall regeneration. The authors proposed that de-esterification of the carboxyl groups of pectin uronic-acid units permits the formation of a gel that envelops the protoplast, and then the rigid cellulose-hemicellulose framework is formed along with this gel matrix.

Soon after this, Emmerling and Seitz [81] isolated a xyloglucan oligosaccharide isolated from the walls of suspension-cultured carrot cells and examined its impact on regenerating carrot protoplasts. This nonasaccharide named XG9 (Glc4Xyl3GalFuc) exhibited anti-auxin properties, whereby XG9 addition in nanomolar concentration to media containing 2,4-D influenced both the viability of the isolated protoplasts and the activity of glycan synthases, with effects similar to those of omitting 2,4-D from the regeneration medium.

Phytosulfokine-α (PSK) is a plant specific disulfated pentapeptide known to be involved in the initial steps of cellular dedifferentiation, proliferation, and re-differentiation, that exhibits a biological function similar to that of plant hormones when added at nanomolar concentrations [82,83]. Since PSK increased the plant regeneration efficiency through somatic embryogenesis and this trait is linked to a thinning of the cell walls [84], it was legitimate to assess whether there were any modifications of the cell wall fractions associated with the onset of the regeneration process. Thus, Godel-Jędrychowska et al. [85] examined the correlation between the fractions (pectin, arabinogalactan protein and extension epitopes) involved in the regeneration of the walls of protoplast-derived cells of carrot and the presence of PSK in the culture medium. They directed various antibodies against the wall components and observed a variable response to PSK in terms of protoplast-derived cell development of the three *Daucus* taxa studied, as well as a diversity in chemical composition of the cell walls between the control and the PSK-treated cultures.

## 9. Protoplasts and Asymmetric Somatic Hybridization in the Family Apiaceae: State-of-the-Art

Generally, desired protoplast fusion could be performed either by chemical or electrical fusion. Due to the charged surface, spontaneous fusion happens very rarely; chemical fusing agents mainly used in the family Apiaceae are 15% Dextran, 10% DMSO, Glycine, 5% to 56% Polyethylene glycol (PEG M.W.1540, 4000, 6000) that have different formula weights, along with osmotic stabilizers such as mannitol or sorbitol. Usually, a mixture of a different combination of compounds such as mannitol, sorbitol, sodium chloride, calcium chloride, calcium nitrate, glycine, sodium hydroxide and buffers is used (see References in Table 2).

**Figure 3:**
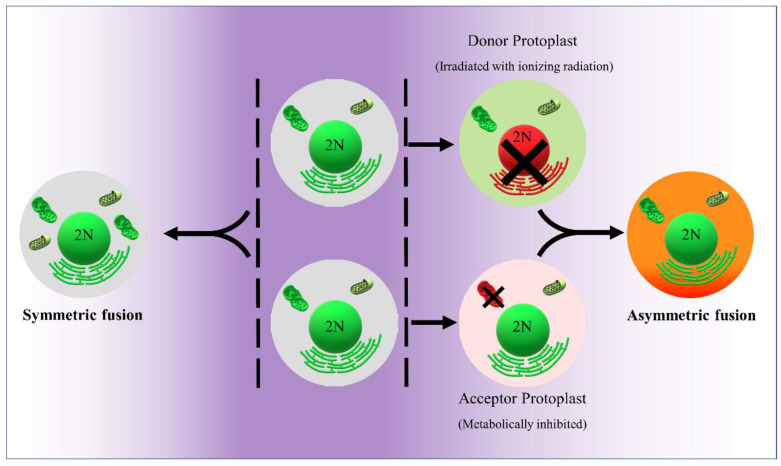
Symmetric and asymmetric protoplast fusion. Donor mitochondria (**Green**), acceptor mitochondria (**Red**).

In 1972, Carlson et al. reported the first successful production of somatic hybrids, of *Nicotiana glauca* and *N. langsdorffii*, showing that organelles transfer is possible and therefore transfer is not limited to genetic material only [86]. Among other strategies available, CMS can be experimentally induced through protoplast fusion and somatic hybridization, which is a technique additively combining the somatic cells from two different genotypes, cultivars, species, or genera aimed at regenerating an entirely novel genotype [87]. In general, a standard somatic hybridization strategy comprises four steps (Figure 3): (a) isolation of parental protoplasts, (b) their chemical or electrical fusion, (c) culture of the heterokaryons obtained to regenerate first hybrid callus then plants, and finally (d) identification and selection of the somatic hybrid lines of interest. Such protoplast fusions may be symmetric, when the contribution of each parent is equivalent (2n = 2x + 2x), or asymmetric when one parent contributed the nuclear and the other the cytoplasmic genetic information (2n = 2x). The latter requires limiting the genetic contribution of one of the parents, for instance by inactivating the nucleus of the donor parent using radioactivity or chemicals (Figure 3) giving rise to a cytoplasmic hybrid or cybrid [88].

Therefore, a cybrid is the type of asymmetric somatic hybrid in which the nuclear genome comes from a single parent whereas the cytoplasmic genomes are inherited from both parents. However, with few exceptions, following cell divisions the chloroplast genome tends to become uniparental while the mitochondrial genome remains recombinant, with segments of both parental mtDNAs [89,90,91,92]. One feature that justifies the appeal of cybrids for breeding programs is the maintenance of cultivar integrity given that the entire nuclear genome comes from one parent [93]. Noteworthy, cybrids are often male-sterile, and allow for the transfer of genomic fragments from wild plants with interesting agronomic traits to commercial crops. Additionally, protoplast fusion permits to sidestep existing barriers to sexual hybridization and hence combine sexually incompatible germplasms between phylogenetically close or distant plants, but also to transfer to a commercial crop those desirable traits that are encoded by the plastid or mitochondrial genomes of an uncultivated genotype.

Recently, Gieniec et al. developed a protocol for the real-time detection of somatic hybrids with stable non-toxic fluorescent protein (FP) tagging of mitochondria during electrofusion using carrot protoplasts [79]. Thus, either cyan (eCFP), green (sGFP), yellow (eYFP) or the mCherry variant of red FP (RFP), with a fused mitochondrial targeting sequence, were introduced to carrot cell lines by *Agrobacterium*-mediated transformation and were subsequently used as a source of protoplasts for electrofusion, after selection to confirm stable labelling. First, the authors assessed the effect of various direct current (DC) parameters on protoplast integrity and on their ability to form heterokaryons. They found that the protoplast response and hybrid cell formation depended on DC voltage and pulse time, and varied among protoplast sources. Heterokaryons (GFP + RFP or YFP + RFP) were identified through their dual-color fluorescence [79]. A similar approach based on dual-color fluorescence was developed many years ago for somatic hybridization in protein legumes [94] using two dyes and non GMO material, but this study using FP stable tagging of mitochondria was the first of its kind in carrot. Three hybrids were produced after the symmetric fusion between carrot root and celery mesophyll protoplasts [95].

## 10. The Production of Cybrid CMS Lines via Protoplast Fusion

As indicated above, cybrids are obtained through the fusion between donor protoplasts, whose nuclei are irradiated by ionizing or non-ionizing radiation treatment such as Gamma-rays, X-rays or Ultraviolet (UV) rays, and recipient protoplasts whose cytoplasm organelles are metabolically inactivated by treatment with chemicals such as iodoacetamide (IOA) or iodoacetate. Ultimately, this leads to the production of a fusion product with an intact cell nucleus and a cytoplasm from recipient and donor, respectively. 

The fusion events are obtained through chemical (PEG-mediated) and/or electrical methods to give somatic hybrids or cybrids. A 15 mM IOA treatment was necessary to achieve 90% true hybrids in the Brassicaceae as although a 7.5 mM IOA treatment given to inactivate *B. oleracea* protoplasts was sufficient, when fused with non-treated *B. campestris* these yielded only 43% true hybrids. This shows the nurse effect that helps treated protoplasts to tolerate even the double dose of IOA needed to achieve 90% true hybrids over a time of 15 min [96]. Bruznican and co-workers increased the exposure time from 20 min to 25 min to overcome such nursing effects at 10 mM IOA [34]. In 2000, Yamamoto et al. reported the conversion of a MS-1 CMS line into a fertile line with iodoacetamide treatment and X-ray irradiation of fertile cultivars, through electrofusion [76]. 

In 1982, Maliga et al. [97] first reported cytoplast-protoplast fusion, where cytoplasts were the cells whose nucleus was removed. Before that, two methods had been successfully devised to obtain such cytoplasts, i.e., either by application of cytochalasin B [98], or by ultracentrifugation using a discontinuous percoll/mannitol gradient [99]. These methods have potential for the transfer of organelle-mediated traits to obtain the desired cybrids [87] but are yet to be explored in carrot. Another, still unexplored approach in the family Apiaceae is cytoplast-protoplast fusion and asymmetric fusion, as symmetric hybridization of such partners also produces cybrids, this being a common phenomenon in a few species like citrus and tobacco. Thus, in tobacco half of the regenerated plants produced by interspecific symmetric somatic hybridization were cybrids [87]. This lack is rather surprising considering that in carrot intergeneric, intraspecific and interspecific somatic hybridization have been well known for many years [55,59,100,101]. 

In this context, Wang et al. [95] regenerated three hybrids after a symmetric fusion between celery mesophyll and carrot root protoplasts, while Tan et al. [77] regenerated 11 petaloid celery CMS plants following the asymmetric fusion between IOA-treated celery and UV-treated carrot protoplasts. Most recently, CMS hybrids were also recovered after the fusion between carrot UV-inactivated donor protoplasts and inactivated acceptor protoplasts of celeriac, while the use of coriander or celery donor protoplasts did not permit the recovery of CMS cybrids in this study [34]. 

The cybridization approach combines the cytoplasm of a donor protoplast of one species with the nucleus of an acceptor protoplast belonging to another species, thus permitting the generation of novel interspecific nucleo-cytoplasmic genomic configurations. The obtained cybrid tissues result, therefore, from an asymmetric protoplast fusion and, on occasions, plants regenerated from them may display CMS due to the incongruent expression of nuclear and cytoplasmic DNA. This was shown in several species in the Brassicaceae with the nuclear genome of the donor partner inactivated by UV [96,102,103] or iodoacetamide (IOA), and rearrangements of the mitochondrial genes [104]. In the Solanaceae, too, alloplasmic CMS has been obtained in *Nicotiana* using X-rays [105,106], while in tomato this was achieved with IOA-treated protoplasts fused with γ or X-ray irradiated protoplasts of potato [107]. Here, the authors generated novel CMS types in tomato after fusing *Lycopersicon esculentum* IOA-treated protoplasts with *Solanum acaule* or *S. tuberosum*. Fertile plants were only recovered when the donor was *S. lycopersicoides*, despite the presence of donor mitochondria fragments in the acceptor protoplasts. This underlined the importance of the donor genotype for the generation of a novel CMS type. 

One example where asymmetric protoplast fusion is used routinely concerns the genus *Citrus*, a model for somatic hybridization. Thus, Grosser et al. [108] transferred CMS from Satsuma mandarin to other seeded cultivars, and Aleza et al. [109] recovered cybrids with different mitochondrial and chloroplast combinations following symmetric fusions between two mandarine genotypes (Chios and Clementine) and between Chios mandarine and Sanguinelli orange.

## 11. The Requisites for a Successful Somatic Hybridization

For any asymmetric protoplast fusion to succeed, the availability of efficient plant regeneration methods is a pre-requisite, particularly for the acceptor species. A second pre-requirement is the reproducibility of a reliable method to inactivate the nuclear DNA from the donor and the cytoplasmic DNA from the acceptor. The existence of a routinely working method for protoplast fusion is needed to create interspecies hybrids. Finally, the cytoplasmic genome characterization of plants regenerated from the fusion events is essential when asymmetric fusions are employed to integrate chloroplast or mitochondria into the acceptor, and the hybridity of shoots regenerated from such fusions is assessed using molecular markers from both the nuclear and cytoplasmic genomes, to identify those regions in the genome of the hybrids that are polymorphic between the donor and the acceptor species. Alternatively, plasmotype discrimination can also be undertaken by high resolution melting analysis based on the presence of SNPs, insertions and deletions (INDELS) or SSRs [110]. 

To date, a number of methodologies for the regeneration of plants from protoplasts have been developed in different species, and there are various techniques with promising applications in breeding, such as increasing the genetic diversity through somaclonal variation, the transfer of cytoplasmic or nuclear genes through somatic hybridization, or the direct introduction of foreign genes through the transformation of isolated protoplasts [109,111,112,113,114]. In this context, the use of protoplast fusion and somatic hybridization for the production of novel hybrids should focus on desirable agricultural traits, aiming at combinations that can only be achieved through protoplast fusion, subsequently using the somatic hybrids for conventional breeding and broadening the range of crops where protoplast technology is exploitable [115]. In turn, this context restricts the potential for the utilization in fusion experiments of protoplasts carrying specific traits that were introduced following genetic transformation. Indeed, they would be considered GMOs and hence would be subject to culture regulations for the somatic hybrids ultimately recovered, and their progenies, in several countries, particularly in the European Union [116,117].

In the 1980s, somatic hybridization was considered as “the” biotechnique with the potential to remodel crop improvement and agricultural research for years to come. However, this assumption is yet to become true, which resulted in a shift of research focus to molecular-based techniques. This failure of a major impact of somatic hybridization on crop development can be ascribed to many reasons. Among them are the difficulty in the isolation and culture of protoplasts added to that encountered for plant regeneration from protoplasts in many crops where it has proved highly genotype-dependent, and also the fact that somatic hybrid plants exhibit a higher ploidy level or chromosome complement since they did not result from a crossing and gamete recombination but from a genome-addition process. Nevertheless, this drawback of using somatic hybridization has not been paramount with citrus where it is presently applied for breeding of novel genotypes [118]. 

## 12. Miscellaneous Studies with Carrot Protoplasts

In an early study, Chen and Wang [119] succeeded in cryopreserving both cell suspensions and protoplasts of carrot by vitrification. Both were precultured in liquid MS medium with 0.175 M sucrose for 3 d followed by 0.4 M sorbitol for 1 d, and they were quenched in liquid nitrogen following loading in 25% PVS2 at room temperature of precultured cells (for 5 min) and protoplasts (for 3 min) and treatment with 100% PVS2 at 0ºC (for 7.5 min and 5 min, respectively). As compared to this, only 47% of the untreated control survived after cryopreservation.

Other studies concerned somaclonal (protoclonal) variation, which is a means to recover novel genotypes with interesting traits, and has been used in many species for this purpose. In carrot, Grzebelus et al. [120] subjected freshly isolated protoplasts and 5-day-old protoplast-derived microcolonies to in vitro selection using culture filtrates from the fungus responsible for black rot disease (*Alternaria radicina*). They revealed that the fungal culture filtrate decreased the viability and plating efficiency of isolated protoplasts and inhibited cell divisions in the microcolonies at all concentrations tested above 5% (*v*/*v*). However, the authors also observed that these responses were genotype-dependent and a few plants which were regenerated with 1%, 2% and 3.5% fungal culture filtrate were hence deemed to be stress tolerant. Noteworthy among such regenerants up to 19% were tetraploids, while only 5% of tetraploid plants were regenerated from the control protoplasts. However, RAPD markers did not reveal any large chromosomal rearrangements between the controls and the regenerants obtained after selection. Some of the plants regenerated from both control protoplasts and cultures selected with the fungal culture filtrate showed a lower susceptibility to *A. radicina* compared to seed-derived plants, but those derived from fungal culture treatments were less capable of flowering and showed a higher rate of male sterility. 

The study above confirmed the potential interest of protoclonal variation, both spontaneous and induced, for the generation of genetic novelties of interest in carrot, and prompted another study by the same team where Kiełkowska et al. [121] undertook experiments on in vitro selection for salt tolerance with carrot protoplast cultures. Thus, they exposed protoplasts of three carrot accessions to increasing concentrations of NaCl up to 400 mM and found that NaCl at 50 mM or more reduced plating efficiency while 200 mM NaCl or higher completely arrested cell division. Thereafter, they subjected the protoplast-derived plants from the control and 50–100 mM NaCl treatments to an 8-week salt stress in greenhouse conditions induced by salinized soil (electrical conductivity, EC, of 3 and 6 mS cm^−1^). They found that the 50 mM NaCl stress in vitro induced polyploidy among regenerants, but also that the plants from the 50 and 100 mM NaCl-treated protoplast cultures had a higher survival rate under salt stress compared to the controls. These salt-stressed plants also accumulated anthocyanins in the petioles, produced denser hairs on the leaves and petioles, and exhibited altered pollen viability and seed setting compared to control plants. 

## 13. The Impact of Artificial Intelligence on Protoplast Technology-Based Approaches in Future

Some of the drawbacks above can be removed by employing state-of-the-art technologies such as artificial intelligence (AI), neuronal networks and robotic platforms. In this respect, the use of computational approaches may have a valuable impact in improving trial and error in regeneration systems [122]. To optimize and develop regeneration protocols, AI models can be taken into consideration. Several studies reported the accuracy and reliability of artificial intelligence methodology to optimize various processes such as callogenesis, protoplast and cell growth, somatic embryogenesis, androgenesis, shoot regeneration, rhizogenesis, hairy root cultures, sterilization, temperature inside the culture containers, plant virus detection, secondary metabolite production, microshoot length, in vitro physiological disorders, shoot organogenesis, in-vitro rooting and acclimatization in several crops [123,124]. Shiotani et al. used Multilayer perceptron (MLP) of artificial neural networks (ANNs) to classify the alive or dead cell status of *Arabidopsis thaliana* cultured protoplasts using digitalized imaged shape and color of cells [125]. In the family Apiaceae, MLP was used for the classification of somatic embryos and non-embryogenic structures of *Apium graveolens* that can be selected for transfer to the next culture phase [126,127]. Likewise, MLP was also applied for cell growth prediction and optimization in order to determine the final biomass level of *Daucus carota* by using initial inoculum and sugar concentration data [128]. AI models could be applied for the emerging field of genetic engineering and genome editing, i.e., via clustered regularly interspaced short palindromic repeats (CRISPR)- CRISPR associated protein 9 (Cas9). Genetic transformation mainly relies on multiple factors such as nutrients, light, temperature and bacterial optical density but not the least on the plant genotype [124]. Recently, robotic platforms were used for isolation of protoplasts, minimizing the time duration compared to agrobacterium mediated or biolistic methods for early detection of transformed plants [129].

Most recently, protoplasts were used for production of transgene-free carrot following PEG-mediated transformation using CRISPR technology, for the centromere-specific Histone H3 (*CENH3*) gene [51]. Protoplasts were also used earlier to verify the blockage of the anthocyanin synthesis in a model purple carrot callus following the knock-out of *F3H* gene to demonstrate successful site-directed mutagenesis in carrot with CRISPR/Cas9 and the usefulness of a model callus culture to validate genome editing systems [130]. In this respect, the new plant breeding technologies (NPBT) approach must be utilized in the species lacking host susceptibility to transformation mediated by *Agrobacterium* [44]. Use of protoplasts for CRISPR/Cas system, artificial intelligence (AI), neuronal networks, and robotic platforms for isolation, transformation and screening of protoplast isolation are emerging fields in agriculture [131].

## 14. Conclusions

The rising population remains challenging due to the increased demand for food, leading to a simultaneous need for high production and yield, coupled with a good quality of crops. The family Apiaceae contributes a major share to the human diet and hence shows a need for higher production. The use of protoplasts for somatic hybridization increased due to commercially important traits that are governed by mitochondria and plastids. To maintain the uniformity in quality and yield, CMS lines are one of the good sources for hybrid seed production. Other traits of agronomic and economic relevance that could benefit from the availability of somatic hybrids in members of the family Apiaceae are those associated with nutrition, disease tolerance and even natural color production. For protoplast fusion, well established isolation, pre-treatment and regeneration from different tissue sources are the primary needs of today. Protoplast fusion has often remained a bottleneck for obtaining hybrids due to the unavailability of nuclear irradiation facilities including Gamma rays and X-rays. As an alternative, the more easily available ultra violet radiation has been used. In addition, fusion efficiency with chemical and electrical fusion needs to be improved. To date, a number of studies have reported somatic hybridization in the family Apiaceae, but the transfer of a trait and its commercialization have remained challenging. In future, use of evolving technologies such as CRISPR and AI will be helpful when associated with protoplast technology.

## Data Availability

No new data were created or analyzed in this study. Data sharing is not applicable to this article.
